# Mitochondrial activity regulates T cell memory, self renewal and anti tumor function in CD8+ T cells

**DOI:** 10.1186/2051-1426-1-S1-O11

**Published:** 2013-11-07

**Authors:** Madhusudhanan Sukumar, Jie Liu, Joseph Crompton, Mahadev Rao, Yun Ji, Toren Finkel, Luca Gattinoni, Nicholas Restifo

**Affiliations:** 1 Center for Cancer Research, National Institutes of Health, Bethesda, MD, USA; 2 Laboratory of Molecular Medicine, National Institutes of Health, Bethesda, MD, USA

## 

The ability of tumor-reactive CD8+ T cells to eradicate tumors following adoptive transfer of autologous tumor-infiltrating lymphocytes correlates with their capacity to proliferate and persist for long periods of time. These qualities are found predominantly in naive and less differentiated memory cells including memory stem cells (TSCM) and central memory cells (TCM), but the metabolic control of differentiation remains unknown. T cell differentiation is characterized by an increase in glycolysis and a rise in mitochondrial metabolism characterized by increased mitochondrial mass, activity and reactive oxygen species generation. We previously demonstrated that inhibition of glycolysis resulted in memory T cell formation and anti-tumor function; however the specific role that mitochondrial metabolism plays in regulating T cell differentiation remains unknown. Using TMRM, a fluorescent dye that measures mitochondrial potential in T cells, we found that mitochondrial membrane potential (MP) and reactive oxygen species in T cells critically control T cell longevity. Cells with lower membrane potential (‘low MP’) had a molecular profile characteristic of stem cell memory precursors and displayed an enhanced ability to enter the memory pool as compared to cells displaying higher mitochondrial potential (‘high MP’) characteristic of short lived effectors. Global metabolomic and functional studies revealed that ‘low MP’ cells exhibited increased levels of intracellular free fatty acid metabolites, increased expression of CPT-1a, a rate limiting enzyme involved in fatty acid oxidation and increased mitochondrial spare respiratory capacity, a metabolic property characteristic of long lived memory T cells. In comparison, ‘high MP’ T cells displayed enhanced lactate production. Most importantly, we observed a 100 fold increase in the frequency of secondary memory CD8+ T cells 300 days after adoptive transfer of 'low MP' as compared to ‘high MP’ T cells. In tumor-bearing mice, ‘low MP’ cells exhibited increased cytokine functionality and resulted in the regression of large, vascularized tumors. Subsequent studies revealed that ‘high MP’ cells displayed increased DNA damage that leads to accelerated senescence, but the ‘low MP’ cells exhibit reduced ROS and DNA repair which allows them to persist longer than the ‘high MP’ cells in vivo following adoptive transfer. Our findings suggest that mitochondrial membrane potential critically controls the differentiation of memory versus effector T cells. Pharmacological interventions augmenting mitochondrial spare respiratory capacity hold great promise to improve T cell based immunotherapy for cancer.

